# Identification of Siglec-1 null individuals infected with HIV-1

**DOI:** 10.1038/ncomms12412

**Published:** 2016-08-11

**Authors:** Javier Martinez-Picado, Paul J. McLaren, Itziar Erkizia, Maureen P. Martin, Susana Benet, Margalida Rotger, Judith Dalmau, Dan Ouchi, Steven M. Wolinsky, Sudhir Penugonda, Huldrych F. Günthard, Jacques Fellay, Mary Carrington, Nuria Izquierdo-Useros, Amalio Telenti

**Affiliations:** 1 AIDS Research Institute IrsiCaixa, Institut d'Investigació en Ciències de la Salut Germans Trias i Pujol (IGTP), Universitat Autònoma de Barcelona, 08916 Badalona, Spain; 2 Institució Catalana de Recerca i Estudis Avançats (ICREA), 08010 Barcelona, Spain; 3 University of Vic-Central University of Catalonia (UVic-UCC), 08500 Vic, Barcelona, Spain; 4 National HIV and Retrovirology Laboratory, Public Health Agency of Canada, Winnipeg, MB, Canada R3E 0W3; 5 Department of Medical Microbiology and Infectious Diseases, University of Manitoba, Winnipeg, MB, Canada R3E 0J9; 6 Cancer and Inflammation Program, Laboratory of Experimental Immunology, Leidos Biomedical Research, Inc., Frederick National Laboratory for Cancer Research, Frederick, Maryland 21702, USA; 7 Institute of Microbiology, University Hospital Center and University of Lausanne, 1011 Lausanne, Switzerland; 8 Department of Medicine, Northwestern University Feinberg School of Medicine, Chicago, Illinois 60611, USA; 9 Division of Infectious Diseases and Hospital Epidemiology, University Hospital Zurich, University of Zurich, 8091 Zurich, Switzerland; 10 Institute of Medical Virology, University of Zurich, 8057 Zurich, Switzerland; 11 School of Life Sciences, École Polytechnique Fédérale de Lausanne, 1015 Lausanne, Switzerland; 12 Swiss Institute of Bioinformatics, 1015 Lausanne, Switzerland; 13 Ragon Institute for MGH, MIT and Harvard, Cambridge, Massachusetts 02139, USA; 14 Genomic Medicine, J. Craig Venter Institute, La Jolla, California 12037, USA

## Abstract

Siglec-1/CD169 is a myeloid-cell surface receptor critical for HIV-1 capture and infection of bystander target cells. To dissect the role of *SIGLEC1 in natura*, we scan a large population genetic database and identify a loss-of-function variant (Glu88Ter) that is found in ∼1% of healthy people. Exome analysis and direct genotyping of 4,233 HIV-1-infected individuals reveals two Glu88Ter homozygous and 97 heterozygous subjects, allowing the analysis of *ex vivo* and *in vivo* consequences of *SIGLEC1* loss-of-function. Cells from these individuals are functionally null or haploinsufficient for Siglec-1 activity in HIV-1 capture and *trans*-infection *ex vivo*. However, Siglec-1 protein truncation does not have a measurable impact on HIV-1 acquisition or AIDS outcomes *in vivo*. This result contrasts with the known *in vitro* functional role of Siglec-1 in HIV-1 *trans*-infection. Thus, it provides evidence that the classical HIV-1 infectious routes may compensate for the lack of Siglec-1 in fuelling HIV-1 dissemination within infected individuals.

## Abstract (web summary)

Binding of virus, HIV-1, to cellular protein Siglec-1 is important for infection of immune cells. Here the authors show that a natural mutation leading to production of truncated Siglec-1 reduces HIV binding and infectivity transfer *in vitro*, but does not substantially affect infection or AIDS outcome in patients.

## Graphical abstract

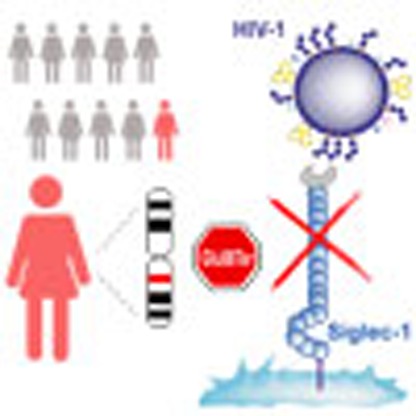

Antigen presenting cells of the myeloid lineage, such as monocytes, macrophages and dendritic cells, initiate immune responses and are crucial to control invading viruses. In the case of HIV-1 infection, however, myeloid cells also promote viral pathogenesis through *trans*-infection of CD4^+^ T cells^1^,^2^. This mechanism involves HIV-1 capture by sialic acid-binding Ig-like lectin 1 (Siglec-1/CD169), a myeloid-cell receptor that recognizes viral membrane gangliosides^3^,^4^,^5^,^6^. Viral capture facilitates the release of trapped viruses at a cell-to-cell contact zone promoting the *trans*-infection of CD4^+^ T cells^7^. Immune activating signals, such as interferon alpha (IFNα) or bacterial lipopolysaccharide, are present throughout the course of HIV-1 infection^8^ and induce Siglec-1 expression on myeloid cells^5^,^6^,^9^. However, under these inflammatory conditions, DC-SIGN and other C-type lectin receptors previously implicated in HIV-1 *trans*-infection^2^,^10^ play a minor role in viral transmission^5^,^6^,^11^. Thus, Siglec-1 is an important inducible receptor that could accelerate HIV-1 transmission in lymphatic tissues, where many T-cells are in contact with myeloid cells. Recent studies performed *in vivo* in mouse models demonstrated that the robust infection of retroviruses in secondary lymphoid tissues requires Siglec-1 and does not rely on the mouse DC-SIGN homolog^12^. Siglec-1 *trans*-infection, cell-free virus infection and cell-to-cell viral transfer between infected and non-infected cells are all important routes of viral dissemination. Yet, the relative contribution of *trans*-infection to HIV-1 transmission and AIDS disease progression remains unknown.

There has been considerable interest in using human genetic diversity to dissect the role of various genes in defence against pathogens *in natura*^13^. Recently, protein-truncating variants have been catalogued in the human genome^14^,^15^,^16^,^17^. These are variants that are likely to disrupt the function of the corresponding allele^18^,^19^. The identification of the CCR5Δ32 variant was pivotal to understanding how human genetic variation contributes to differences in susceptibility to HIV-1 infection^20^, equally affecting all routes of viral spread.

Here, we aimed to identify *SIGLEC1* null individuals to dissect the specific contribution of *trans*-infection to HIV-1 pathogenesis *in vivo*. We find individuals with a specific loss-of-function variant in *SIGLEC1* gene that completely abrogates Siglec-1 receptor expression on primary monocytes and their capacity to *trans*-infect HIV-1 *ex vivo*. Despite this *ex vivo* phenotype there is a striking absence of marked differences in HIV-1 acquisition or clinical evolution of individuals carrying *SIGLEC1* loss-of-function alleles.

## Results

### Genetic description of SIGLEC1 variants

**Figure 1:**
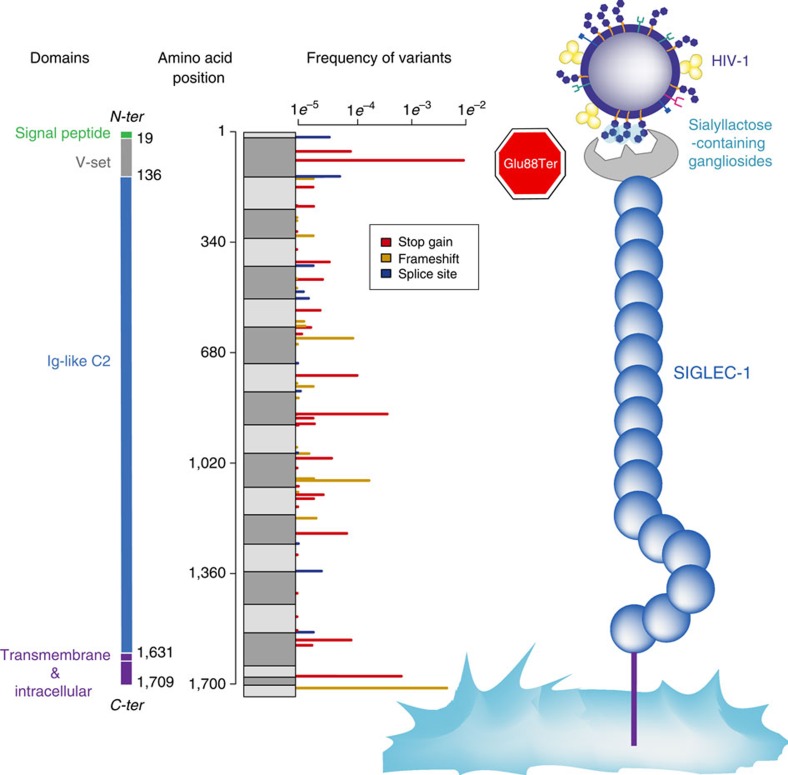
Location and frequency of *SIGLEC1* protein truncating variants. Protein domains are represented in different colours. Data from the Exome Aggregation Consortium (exac.broadinstitute.org) identifies 70 protein truncating variants in *SIGLEC1* including 33 stop gain (red), 24 frameshift (yellow) and 12 splice disrupting (blue) variants. Grey boxes indicate amino acid blocks encoded by each exon. With the exception of Glu88Ter, all protein truncating variants occur at <1% frequency. Glu88Ter is located in the V-set domain of Siglec-1, the region that recognizes sialyllactose in HIV-1 membrane gangliosides.

We used data from the Exome Aggregation Consortium (ExAc.broadinstitute.org) to identify naturally occurring knockout mutations in *SIGLEC1*. In that sample of ∼63,000 individuals we observed 70 protein truncating variants, that is, stop-gain, frameshift or splice site (Fig. 1). With the exception of rs150358287, a stop-gain variant resulting in an early stop codon at amino acid position 88 (Glu88Ter), truncating variants are of very low frequency (<1%). The Glu88Ter variant occurs in the second exon of *SIGLEC1* (C to A transversion at position 3706494 on chromosome 20, GRCh 38 build reference sequence) and is predicted to truncate both major transcripts of *SIGLEC1*. The stop-gain allele is found at highest frequency in individuals of European and South Asian ancestry (∼1.3%) and is rare or absent in African and East Asian populations (<0.5%).

To assess the frequency distribution of this polymorphism in an HIV-1-infected population, we combined genotype data from exome sequencing (*n*=392), exome chip (*n*=2,212) and direct genotyping (*n*=1,129) in participants of the Swiss HIV Cohort Study (SHCS). In 3,733 individuals whose clinical characteristics are detailed in Table 1 (95% reported European ancestry), we observed 85 Glu88Ter heterozygotes and 2 homozygotes for the stop-gain variant (allele frequency=1.2%). Thus, we identified individuals in whom to assess the consequences of Siglec-1 haploinsufficiency and knockout *in vivo* and *ex vivo*.

### Functional analysis of *SIGLEC1* null variant

**Figure 2:**
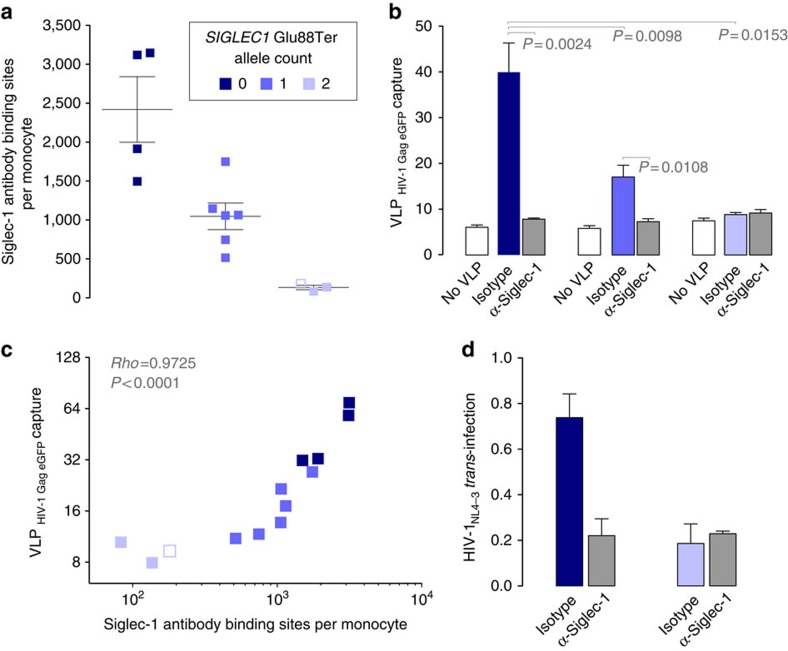
Siglec-1 expression and *trans*-infection across distinct *SIGLEC1* genotypes. Monocytes were isolated and cultured 24 h in the presence of 1,000 U ml^−1^ of IFNα to induce Siglec-1 expression. (**a**) Quantification of Siglec-1 expression levels assessed by flow cytometry. Empty box represents a repeat analysis of one Siglec-1 null homozygote. (**b**) Capture of fluorescent HIV-1 VLPs by monocytes from distinct genotypes previously exposed to isotype or α-Siglec-1 mAbs. Geometric mean fluorescence intensity of monocytes not exposed to VLPs is also depicted to show the background levels of the assay (empty bars). (**c**) Correlation between Siglec-1 expression levels and viral capture values of isotype-treated monocytes. (**d**) HIV-1 transmission to a reporter CD4^+^ cell line from monocytes of opposing homozygous individuals pre-incubated with isotype or α-Siglec-1 mAbs. HIV-1 infection of reporter cells was determined by induced luciferase activity. Data show mean relative light units and SEM of cells from two homozygous individuals with the common allele and one Siglec-1 null homozygote.

To confirm that Siglec-1 Glu88Ter homozygous individuals truly lack receptor expression, we performed functional assays with cryopreserved cells collected from individuals with all three possible genotypes. We induced Siglec-1 expression in isolated monocytes using IFNα and determined the absolute number of Siglec-1 antibody binding sites per monocyte (Fig. 2a). Compared to individuals homozygous for the common allele, heterozygous individuals expressed approximately half of the amount of protein observed, while null individuals showed only background expression levels (Fig. 2a). Next, we analyzed the ability of monocytes to capture fluorescent HIV-1 virus-like particles (VLPs) displaying specific gangliosides that are efficiently recognized by Siglec-1 (refs 3, 4). IFNα-activated monocytes from individuals with the common allele showed the highest viral capture capacity followed by heterozygous and then by null individuals (Fig. 2b), which captured only residual levels of VLPs. To investigate whether this binding was specific for Siglec-1, cells were pre-treated with a monoclonal antibody (mAb) against Siglec-1. Treatment led to a significant reduction of VLP uptake in monocytes from common allele and heterozygous individuals (Fig. 2b), while it had no inhibitory effect on *SIGLEC1* null individuals. The VLP uptake of monocytes from distinct *SIGLEC1* genotypes strongly correlated with the mean number of Siglec-1 antibody binding sites per cell (Fig. 2c). To assess the general HIV-1 transfer capacity of Siglec-1 compared to other possible receptors on IFNα-activated monocytes from homozygous individuals, we pulsed cells with equal amounts of infectious HIV-1_NL4-3_ in the presence or absence of blocking mAbs and co-cultured them with a CD4^+^ reporter cell line (Fig. 2d). Monocytes from individuals with the common allele had higher capacity to *trans*-infect than did *SIGLEC1* null cells (Fig. 2d). *Trans*-infection was inhibited with a mAb against Siglec-1, which had no blocking effect on *SIGLEC1* null monocytes (Fig. 2d). Overall, these results indicated that *SIGLEC1* null individuals lack functional Siglec-1 expression and HIV-1 *trans*-infection capacity, ruling out genetic compensation mechanisms or a possible stop codon read-through that could alleviate the null status^21^.

### *SIGLEC1* null variant in HIV-1 acquisition

We next tested for an impact of the Glu88Ter variant on HIV-1 acquisition. Given the population frequency differences at which this variant occurs, we limited these analyses to 3,558 individuals of European ancestry to prevent confounding. The working hypothesis is that if Siglec-1 were essential for infection, homozygous Glu88Ter would not be infected—at least via mucosal exposure. The observation of two HIV-1-infected Glu88Ter homozygotes ruled out a requirement for a functional Siglec-1 protein in HIV-1 acquisition (that is, it does not mirror the CCR5Δ32 effect regarding R5-tropic virus infection). In addition, the observed frequency of the Glu88Ter allele in the HIV-1-infected population (1.2%) is nearly identical to the frequency in Europeans from the ExAc sample (1.3%) and does not differ depending on route of infection (1.15% parenteral, 1.2% sexual, *P*=0.95). Taken together, these results suggest that functional *SIGLEC1* is not required for HIV-1 acquisition regardless of route of exposure.

### *SIGLEC1* null variant in HIV-1 progression

**Figure 3:**
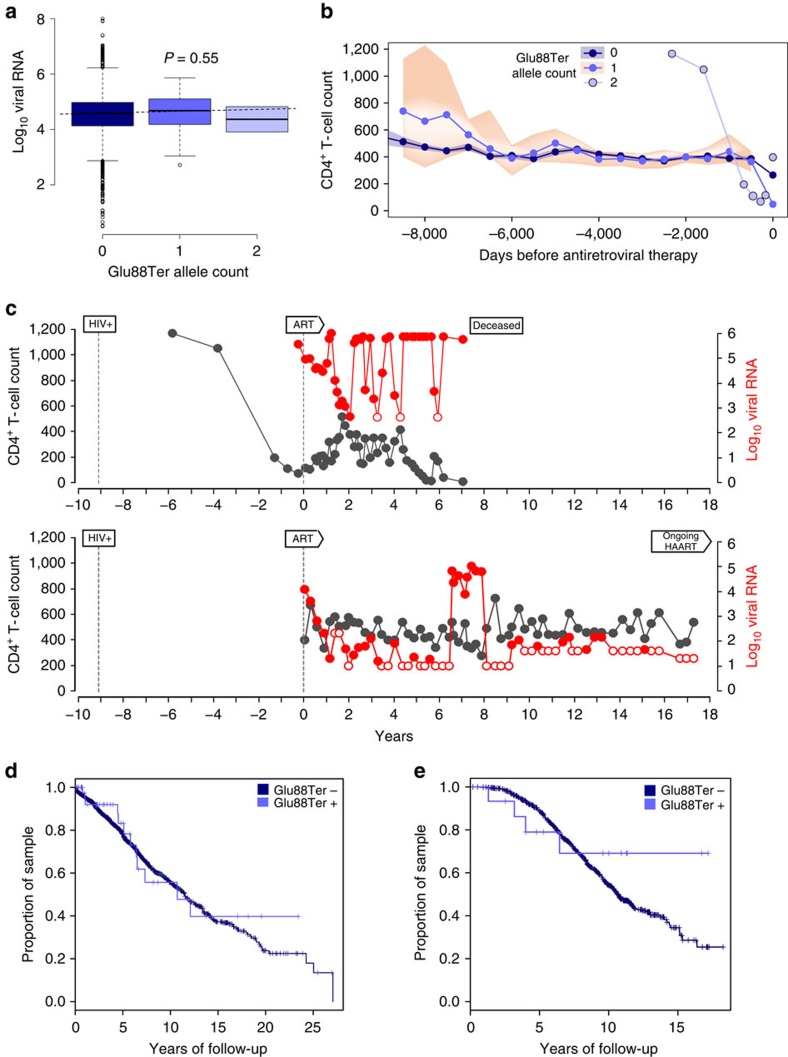
Analysis of association between Siglec-1 Glu88Ter and HIV-1 clinical outcomes in the absence of antiretroviral treatment. (**a**) Set point viral load of individuals from the SHCS with or without the Glu88Ter allele (*n*=2,243). (**b**) CD4^+^ T-cell count dynamics of individuals from the SHCS with or without the Glu88Ter allele (*n*=3,385). CD4^+^ T-cell counts (cells mm^−3^) were binned using 500 days windows, counting backwards from the date of antiretroviral treatment start or loss of follow-up. Median CD4^+^ T-cell values (lines) and interquartile ranges in each bin (shaded areas) are shown for individuals carrying no copies (*n*=3,305) or one copy (*n*=78) of the Siglec-1 Glu88Ter allele. Actual CD4^+^ T-cell values are shown for the two Siglec-1 Glu88Ter homozygotes. (**c**) Plasma viral RNA level (cp ml^−1^) and CD4^+^ T-cell count (cells mm^−3^) dynamics of the two Siglec-1 Glu88Ter homozygotes. The dates of first HIV-1 positive report and of ART initiation are depicted. Open circles indicate negative values bellow the represented detection level. (**d**) Time to AIDS measured in the SHCS cohort (*n*=2,458 common allele Glu88; *n*=53 Glu88Ter including 52 heterozygous and 1 homozygous individuals) or (**e**) the MACS cohort (*n*=401 common allele Glu88; *n*=12 Glu88Ter which were all heterozygous).

We assessed the potential impact of the *SIGLEC1* null variant on HIV-1 viral load and disease progression. As shown in Fig. 3, the presence of one or two copies of the Siglec-1 Glu88Ter allele had no impact on plasma set point viral load (*n*=2,243; Fig. 3a). Similarly, there was no difference in CD4^+^ T-cell dynamics (*n*=2,302; Fig. 3b) or in CD4^+^ T-cell nadir. We also investigated the disease course (viral RNA level and CD4^+^ T-cell counts) for the two homozygous Glu88Ter individuals, which supported viral replication (Fig. 3c). Finally, we assessed the impact of the Glu88Ter variant on progression to AIDS (as defined in 1987) in the SHCS (Fig. 3d), where we analyzed 52 Glu88Ter heterozygous and 1 homozygous individuals out of 2,511 individuals. We performed the same analysis in an independent cohort (the Multicenter AIDS Cohort Study, MACS) after genotyping 413 Caucasian HIV-1-infected individuals with documented seroconversion date (Fig. 3e). In the MACS cohort, we identified 12 heterozygous individuals, but no homozygous Glu88Ter individuals were found. Although there was a slow progression to AIDS observed in both the SHCS and MACS cohorts for Glu88Ter individuals, it did not reach statistical significance.

## Discussion

The identification of two homozygous HIV-1-infected individuals with a confirmed loss-of-function variant in *SIGLEC1*, both of whom were infected via mucosal exposure, indicates that Siglec-1 *trans*-infection is not indispensable to establish HIV-1 infection through sexual contact. Thus, Siglec-1 independent mechanisms of infection are sufficient to support mucosal HIV-1 spread. In the absence of *trans*-infection, the classical HIV-1 infectious routes, including cell-free virus infection or cell-to-cell HIV-1 transmission, compensate for the lack of Siglec-1 and are able to fuel HIV-1 dissemination within infected individuals. This explains why we did not identify marked differences in clinical evolution of individuals with one or two copies of the Siglec-1 Glu88Ter allele.

Given the available sample size of this study and the low Glu88Ter frequency, we can only rule out a large effect of this allele on disease progression. Power simulations indicate that we would need >10,000 samples to detect a relative risk of 5 (similar to the effect of B*57:01 on HIV-1 control) at *P*<0.05 under a recessive model. This sample size far exceeds even the largest genome-wide studies of HIV-1 progression that comprises ∼6,000 patients^22^, which unfortunately does not genotype the Glu88Ter variant and cannot be used to accurately impute the presence of this rare allele. In addition, given that the proposed effect (if any) requires long-term follow-up off therapy (>10 years) it is extremely unlikely that a sufficient sample size could be reached to assess the long-term consequences of this variant on HIV-1 disease. However, the identification of the functional consequences of the Glu88Ter allele might encourage future genetic studies to include this variant and complement our analyses.

These results in humans are in contrast to those observed in the BLT humanized mouse model, where Siglec-1 blockade significantly lowered HIV-1 infection of splenocytes^12^. Discrepancies could be attributed to the fact that our study mainly focused on heterozygous Siglec-1 Glu88Ter individuals, which maintain partial receptor expression and function. Yet, the clinical course of the two homozygous individuals did not show any obvious benefit of absence of Siglec-1. Alternatively, differences with the murine model could be due to the distinct timeframes analyzed in each case. The murine study was limited to the early dynamics of HIV-1 infection^12^, a phase that is missing from the clinical records of most patients, including the two null-homozygous individuals identified here. Hence, it will be important to determine if the lack of Siglec-1 in humans impacts early viral kinetics. If that is the case, current antiretroviral treatments implemented early after infection could include Siglec-1 blocking agents to tackle multiple infectious routes simultaneously and limit the settlement of viral reservoirs. Importantly, the identification of *SIGLEC1* null individuals *in natura* indicates that Siglec-1 could represent a safe therapeutic target.

The analysis of loss-of-function variants serves to illuminate *in vivo* mechanisms of disease. Recent whole-genome and -exome sequencing initiatives indicate that protein truncating alleles can be identified in a large proportion of human genes. We have estimated that up to 85% of all human genes can be observed carrying heterozygous variants^17^. There is strong evidence that heterozygous loss-of-function in innate immunity genes is not compensated in the majority of cases^16^. Thus, the discovery and characterization of human protein truncating variants serves as a general model for the analysis of other human genes involved in pathogen containment.

## Methods

### Patients

The Swiss HIV Cohort Study is an ongoing observational longitudinal study enroling HIV-1-infected individuals since 1988 in Switzerland. Demographic, route of transmission, clinical and laboratory data are systematically collected, and plasma and cells are stored longitudinally every 6–12 months. To date, >19,000 patients have been enroled. At least 50% of all HIV-1-infected, 72% of all AIDS cases in Switzerland are enroled^23^. The MACS^24^ is an ongoing prospective study of the natural and treated histories of HIV-1 infection in homosexual and bisexual men conducted by sites located in Baltimore, Chicago, Pittsburgh and Los Angeles. A total of 6,972 men have been enroled. Clinical characteristics of the Glu88Ter homozygous include a female diagnosed in 1987 that died in 2003 of *Pneumocystis jirovecii* infection and refused treatment. The last CD4^+^ T-cell count was 8 cells mm^−3^ and the last viremia recorded for this individual was >750,000 cp ml^−1^. In 1994, she had an episode of pulmonary tuberculosis. The second Glu88Ter homozygous is a male that was diagnosed HIV+ in 1987 and registered in the SHCS in 1996, when he initiated antiretroviral treatment while clinically asymptomatic. He has been on antiretroviral treatment for most of the past 20 years (except for a short period in 2003 and 2004), with stable CD4^+^ T-cell count between 400 and 600 cells mm^−3^. Last clinical values (recorded in October 2015) were a CD4^+^ T-cell count of 602 cells mm^−3^ (23%) and a viremia <20 cp ml^−1^.

### Ethics statement

The institutional review board on biomedical research from Hospital Germans Trias i Pujol approved this study. Participants of the Swiss HIV Cohort Study and the multicenter AIDS Cohort Study (MACS) consented to the cohort study and genetic analyses, as approved by the corresponding local Ethics Committees.

### Primary cell culture

Frozen peripheral blood mononuclear cells were obtained from HIV-1 patients of the Swiss HIV Cohort Study. Monocyte populations were isolated using CD14 magnetic beads (Miltenyi Biotec) and cultured 24 h with 1,000 U ml^−1^ of IFNα (Sigma) in RPM1 with 10% of heat-inactivated fetal bovine serum (Invitrogen).

### Siglec-1 surface expression analysis by FACS

IFNα-activated monocytes were blocked with 1 mg ml^−1^ of human IgG (Baxter, Hyland Immuno) and stained with 1/10 dilution of α-Siglec-1-PE 7–239 mAb (AbD Serotec) following manufactures instructions at 4 °C for 20 min. Samples were analyzed with FACSCalibur (Becton-Dickinson) using CellQuest software to evaluate collected data. The mean number of Siglec-1 Ab binding sites per cell was obtained with a Quantibrite kit (Becton-Dickinson) as previously described^5^.

### VLP capture and HIV-1 *trans*-infection assays

VLP_HIV-Gag-eGFP_ and HIV-1_NL4-3_ were obtained by calcium phosphate transfection of previously described plasmids^5^. IFNα-activated monocytes (2 × 10^5^) were pre-incubated at 16 °C for 30 min with 10 μg ml^−1^ of α-Siglec-1 mAb (7–239, AbSerotec) or IgG1 isotype control mAb (107.3, BD Bioscience). Capture experiments were performed pulsing monocytes with 150 ng of VLP_HIV-Gag-eGFP_ Gag for 3 h at 37 °C. After extensive washing, monocytes were acquired by FACS. *Trans*-infection assays were performed pulsing monocytes with 300 ng of HIV-1_NL4-3_ for 4 h at 37 °C. After extensive washing, monocytes were co-cultured in duplicate at a ratio of 1:1 with the TZM-bl CD4^+^ target cell line. Cells were assayed for luciferase activity 48 h later (BrightGlo Luciferase System; Promega) in a Fluoroskan Ascent FL luminometer (Thermo Labsystems).

### Genetic analyses

For 392 participants from the SHCS, we captured and sequenced all coding exons using the Illumina Truseq 65 Mb enrichment kit and the Illumina HiSeq2000. Sequences were aligned to the human reference genome version 19 (GRCh 37) using BWA. Variant calling was performed using the HaplotypeCaller module of the Genome Analysis Toolkit version 3.1–1. Only variants passing the variant quality score recalibration thresholds were maintained for further analysis. Genotype data for 2,212 individuals were obtained using the Illumina Infinium Human Exome BeadChip. Direct genotyping for rs150358287G>T in 1,129 individuals from the SHCS and 425 individuals from the MACS was performed by Taqman allelic discrimination using a custom design assay from Applied Biosystems (AHKAY5K). Exome sequencing results were confirmed by PCR and direct sequencing (forward primer: 5′ AGGACGTGCAGGGTGTGAAG33′; reverse primer: 5′ GCTGGAACAGAGGCTGAGAC 3′; annealing temperature: 62 °C; expected fragment: 455 bp).

### Statistical analysis

Statistics of VLP functional assays were performed using unpaired and paired *t* test (considered significant at *P*≤0.05) or the Spearman correlation with GraphPad Prism v.5 software. Set point viral load was calculated as the average of at least three measurements obtained during the chronic phase of infection (minimum 6 months after infection and before the initiation of antiretroviral therapy (ART)). A comparison of variance in set point viral load between genotype groups showed no significant differences (F test *P*=0.18). Association between viral RNA level and genotype was tested using linear regression. Rate of disease progression was measured using all available CD4^+^ T-cell counts beginning at enrolment or estimated date of infection (if known) before the initiation of anti-HIV therapy. The impact of genotype on disease progression was tested using the Cox proportional hazards models.

### Data availability

The clinical and genetic data that support the findings of this study are available on request from the corresponding authors; these data are not publicly available due to information that could compromise research participant privacy. The authors declare that all other data supporting the findings of this study are available within the article or from the corresponding authors upon request.

## Additional information

**How to cite this article:** Martinez-Picado, J. *et al.* Identification of Siglec-1 null individuals infected with HIV-1. *Nat. Commun.* 7:12412 doi: 10.1038/ncomms12412 (2016).

## Figures and Tables

**Table 1:** Clinical characteristics of the SHCS cohort.

	Glu88Ter (homozygous and heterozygous)	Glu88 (homozygous)	
Male *n*; (%)	65 (74.7%)	2835 (77.7%)	
Female *n*; (%)	22 (25.3%)	808 (22.3%)	
Age median; (IQR)	46 (41–50)	47 (41–53)	
Caucasian *n*; (%)	80 (94.1%)	3447 (95.3%)	
Peak viremia median; (IQR)	168,423 (42,036; 440,051)	103,094 (31,314; 290,000)	
CD4 nadir median; (IQR)	236 (118; 306)	225 (121; 323)	
*Mode of HIV-1 acquisition n; (%)*			
Heterosexual	25 (29.4%)	1,116 (30.7%)	
Homosexual	39 (45.9%)	1,617 (44.7%)	
Intravenous drug user	20 (23.5%)	764 (21.1%)	
Other/unknown	1 (1.2%)	120 (3.3%)	

IQR, interquartile range.
